# Free tissue transfer for pyoderma gangrenosum: A case report

**DOI:** 10.1016/j.jpra.2026.09.008

**Published:** 2026-09-13

**Authors:** Ethan A. August, Christopher A. Beaudoin, Rebecca Shirley, James K-K Chan

**Affiliations:** a School of Medicine and Biomedical Sciences, University of Oxford, John Radcliffe Hospital, Headington, Oxford, OX39DU, United Kingdom; b Department of Plastic Surgery, Stoke Mandeville Hospital, Buckinghamshire Healthcare NHS Trust, HP21 8AL, United Kingdom; c Nuffield Department of Orthopaedics, Rheumatology and Musculoskeletal Sciences, University of Oxford, United Kingdom

**Keywords:** Pyoderma gangrenosum, Free flap, Microsurgery, Reconstruction, Case report

## Abstract

Pyoderma gangrenosum (PG) is a rare neutrophilic dermatosis characterised by painful progressive skin ulceration. Medical management with corticosteroids and other immunomodulatory agents remains the mainstay of treatment. Surgical intervention has traditionally been avoided because of the risk of pathergy, whereby minor trauma progresses to ulceration. However, extensive or limb-threatening defects may necessitate reconstruction. We present a case of PG affecting the dorsum of the foot requiring free tissue transfer.

## Introduction

Pyoderma gangrenosum is an uncommon neutrophilic dermatosis characterised by painful nodules or pustules that rapidly progress to ulceration. Lesions are most common on the lower extremities and may develop spontaneously or following minor trauma.^1^ The condition is rare, affecting about 10 people per 100,000.^2^ Although the exact pathogenesis remains unclear, dysregulated neutrophil function and aberrant immune responses are believed to play central roles.

Current guidelines for PG emphasize the importance of accurate diagnosis and gentle, non-adhesive wound care. Medical management for PG includes topical corticosteroids,^3^ escalating to systemic immunosuppressants.^4^

The majority of literature on surgical reconstruction in PG focuses on skin grafting.^5^ Because free tissue transfers are much more invasive, the risk of pathergy is greater. For this reason they have long been avoided in PG. Our case report documents a free tissue transfer for PG, in accordance with STROBE guidance.

## Case presentation

A 58-year-old man presented with a 6 week history of a painful, non-healing ulcer and dysaesthesia over the dorsum of his right foot. Three courses of outpatient antibiotics had not improved his symptoms. Significant medical history included ischemic heart disease, gout, and Raynaud’s phenomenon affecting both feet.

Clinical examination demonstrated a 2 × 2 cm painful ulcer surrounded by blistered skin with a violaceous necrotic margin. Pulses were palpable and capillary refill time was normal. A full blood count was normal. An autoimmune screen showed weakly positive ANCA (1:20) but was otherwise insignificant.

**Fig. 1:**
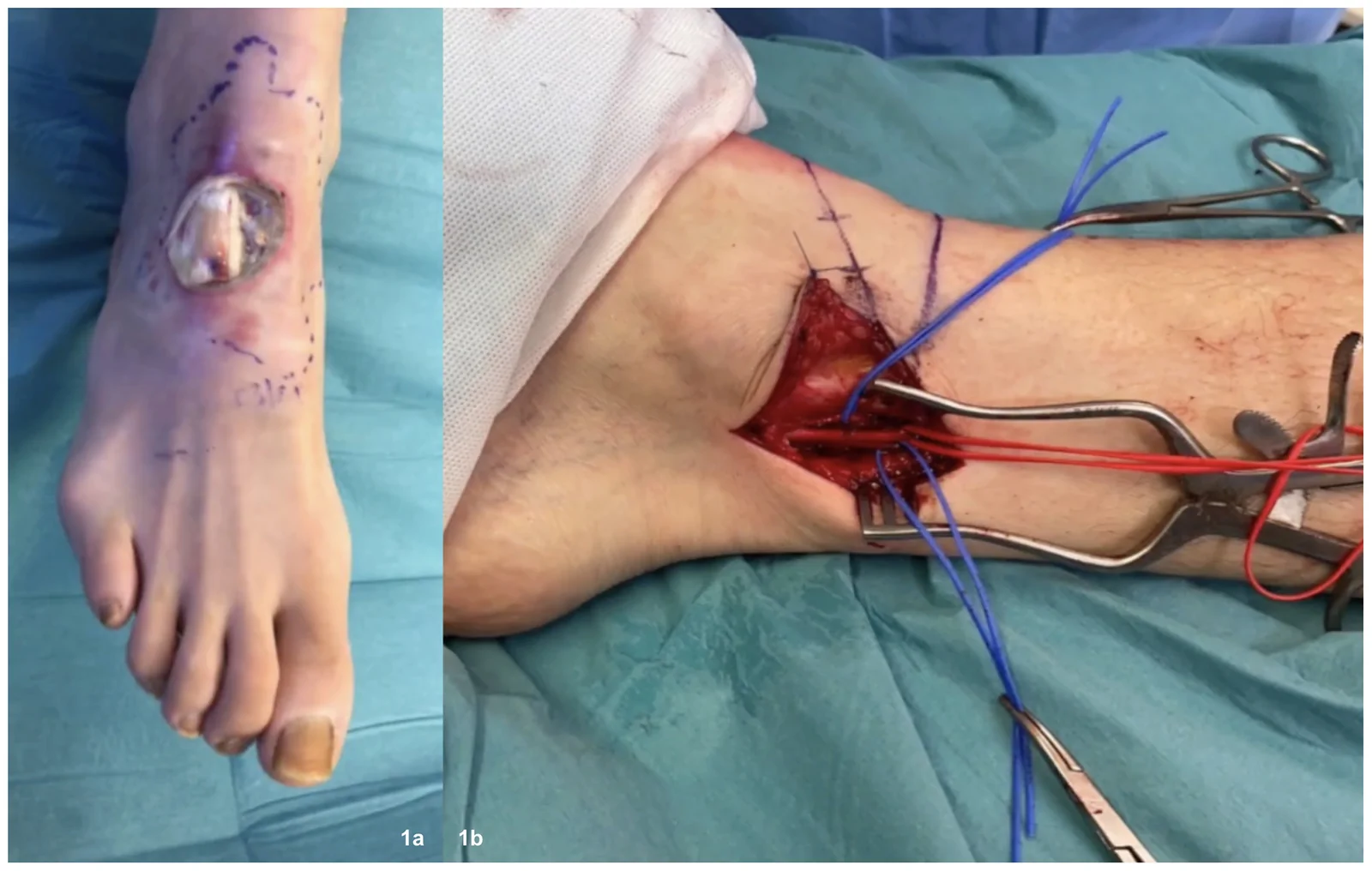
Skin graft failure (1a) and intra-operative free flap reconstruction (1b).

A full thickness skin graft under local anesthesia was initially successful. Within ten days, however, the skin graft failed and the ulcer progressed to 4 × 4 cm (Fig. 1a). Tendons were intact but exposed. Biopsy of the ulcer edge demonstrated marked neutrophilic infiltration and necrotic debris within the dermis, as well as a granuloma in deeper tissues. Microbiological cultures were negative.

The team suspected pyoderma gangrenosum, and decided to perform a free flap reconstruction immediately given the patient’s stable condition and depth of the ulcer. The wound was re-excised and covered with a free anterolateral thigh (ALT) flap, creating an end-to-side arterial anastomosis to the posterior tibial artery and end-to-end venous anastomoses with the venae comitantes (Fig. 1b) Two days post-operatively, the patient commenced prednisolone (40 mg OD), lymecycline (408 mg OD), topical tacrolimus, and cyclosporine (100 mg BD).

Ten days post op, examination showed slow wound healing with gaps in between sutures and wound dehiscence. The patient was brought back for a repeat closure of the ulcer under local anaesthesia. Cyclosporine was increased to 150 mg BD.

**Fig. 2:**
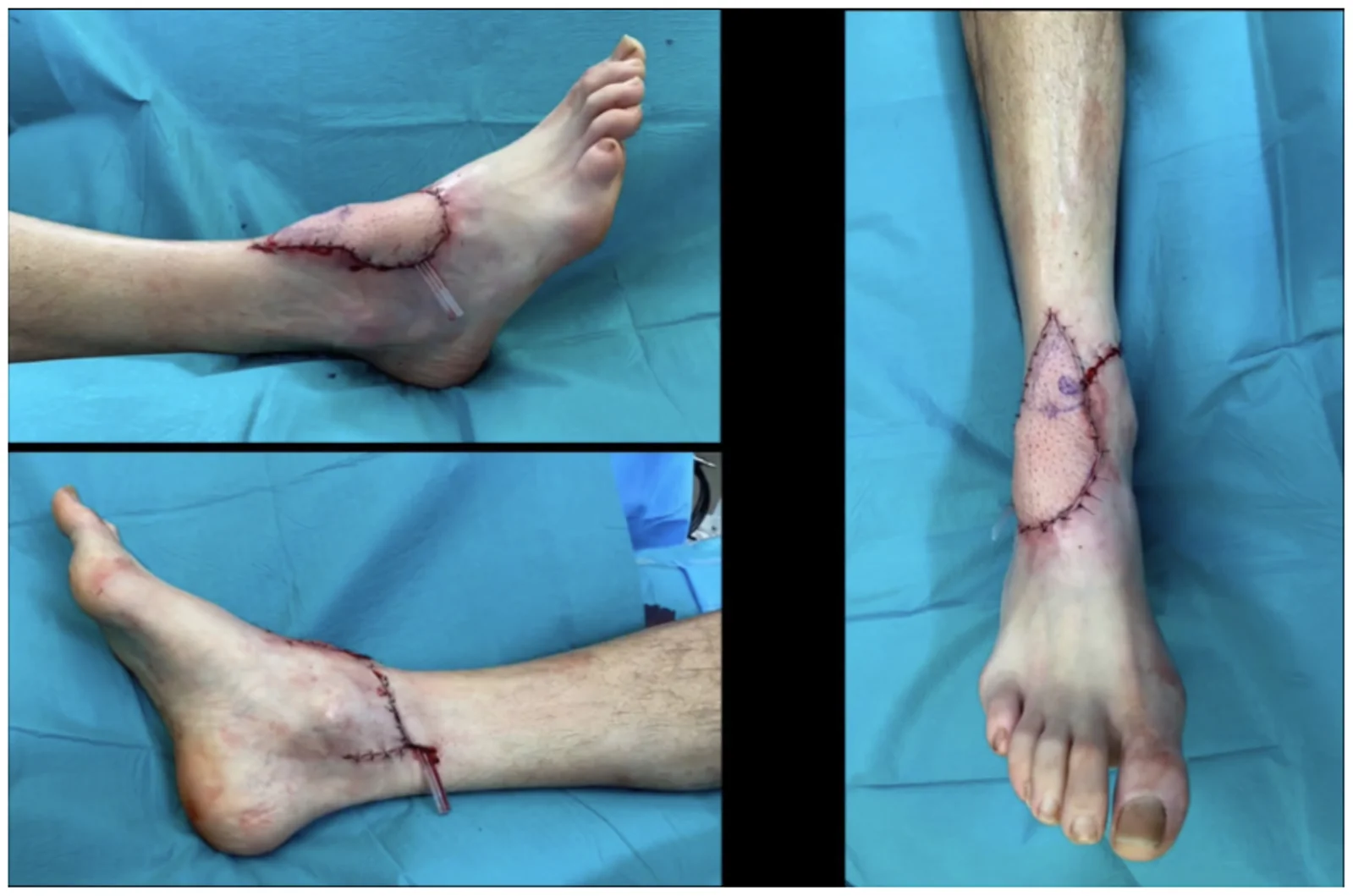
Flap healing 6 weeks post-operatively.

Ten days later, pain had largely subsided and epithelization was observed. The wound was completely healed within 6 weeks. Steroids were discontinued and cyclosporine tapered. The patient returned to independent ambulation at 6 weeks and resumed recreational activities, including long-distance road cycling (Fig. 2).

## Conclusions

Microsurgical free tissue transfer is a viable reconstructive option for non-healing ulcers secondary to PG. However, medical and surgical management must be integrated. Furthermore, operative intervention should be reserved for patients with stable disease and life or limb-threatening ulceration.

## Funding

None.

## Ethical approval

None required.

## Declaration of competing interest

Author James Chan is a Deputy Editor of JPRAS.
